# Oral dydrogesterone as an adjunctive therapy in the management of preterm labor: a randomized, double blinded, placebo‐controlled trial

**DOI:** 10.1186/s12884-021-03562-6

**Published:** 2021-01-28

**Authors:** Suparudeewan Thongchan, Vorapong Phupong

**Affiliations:** Placental Related Diseases Research Unit, Department of Obstetrics and Gynecology, Faculty of Medicine, Chulalongkorn University, Rama IV Road, 10330 Pathumwan, Bangkok, Thailand

**Keywords:** Dydrogesterone, Preterm labor, Latency periods, Progesterone

## Abstract

**Background:**

Preterm birth is a major challenge in obstetric and perinatal care. It is the leading cause of neonatal death. The primary aim of this study was to evaluate the efficacy of oral dydrogesterone on latency period in managing preterm labor. The secondary aims were to evaluate the gestational age at delivery, percentage of preterm delivery before 34 weeks and 37 weeks, time to recurrent uterine contraction, pregnancy outcomes, neonatal outcomes, compliance and side effects.

**Methods:**

This was a randomized, double blinded, placebo-controlled trial. Forty-eight pregnant women with preterm labor, singleton pregnancy, and gestational age of 24–34 weeks were enrolled into the study. The study group received 10 mg of oral dydrogesterone three times per day and the control group received placebo. All pregnant women received standard treatment with tocolytic and antenatal corticosteroids.

**Results:**

The median latency periods were not significantly different between the dydrogesterone group (27.5 days) and placebo group (34 days, *p* = 0.45). Additionally, there were no differences in the gestational age at delivery, percentage of preterm delivery before 34 weeks and 37 weeks, pregnancy outcomes, neonatal outcomes, compliance and side effects. However, the time to the recurrence of uterine contractions in participants that had recurrent preterm labor was longer in the dydrogesterone group than in the placebo group (30.6 ± 12.3 vs 13.7 ± 5.0 days, *p* = 0.01).

**Conclusions:**

Adjunctive treatment with 30 mg of oral dydrogesterone could not prolong latency period in preterm labor when compared to placebo.

**Trial registration:**

ClinicalTrials.gov (Clinical trials registration: NCT 03935152, registered on May 2,2019).

## Background

Preterm birth is a major challenge in obstetric and perinatal care. It is the leading cause of neonatal death and second most common death among children under the age of 5; it is responsible for 1 million deaths each year [1]. Approximately 15 million preterm neonates are born every year, and this number is increasing worldwide [2]. Preterm neonates from early preterm births are at increased risk of morbidity and mortality attributed to immaturity of multiple organs resulting in respiratory distress, cerebral palsy, and vision and neurodevelopment impairment [3]. Preterm birth is defined as birth between 20 0/7 weeks of gestation and 36 6/7 weeks of gestation. The diagnosis of preterm labor generally is based on the clinical criteria of the regular uterine contractions accompanied by a change in the cervical dilation, effacement or both [4]. To prevent preterm birth, various trials have investigated the use of progestogen compounds [5].

American College of Obstetricians and Gynecologists(ACOG) 2012 recommended using prophylactic progesterone supplement in women with a history of preterm birth and in women with a short cervical length to prevent preterm birth [6]. Nevertheless, the role of progesterone supplement remains an area of debate for use in singleton woman with preterm labor but without history of preterm birth.

Multiple trials have examined the use of progesterone supplement in preterm labor patients. Some research found that progesterone maintenance therapy after successful tocolysis significantly prolonged the latency period [7–10], while other studies did not [11, 12]. However, in vitro studies that have investigated the use of various progesterone showed that only dydrogesterone had a rapid and direct inhibition of myometrial contraction and this inhibitory effect was dependent on the dose and time when dydrogesterone was administered [13].

Dydrogesterone, a stereoisomer of progesterone, binds almost exclusively to the progesterone receptor. Dydrogesterone has a good safety and tolerability profile. It is structurally and pharmacologically similar to natural progesterone and has a good oral bioavailability with few side effects, no antiandrogenic or antiestrogenic effect. Its half-life is 5–7 hours [14].

In a previous study, 20 mg per day of dydrogesterone could not prolong latency periods in preterm labor [11]. The dose of dydrogesterone is insufficient because of its half-life. It is possible that at a higher dose of dydrogesterone may be able to prolong the latency period in preterm labor. Therefore, the primary objective of the study was to assess whether 10 mg of dydrogesterone administered three times per day could prolong the latency period in preterm labor. The secondary aims assessed the gestational age at delivery, percentage of delivery before 34 and 37 weeks, time to the recurrence of uterine contractions, pregnancy outcomes, neonatal outcomes, compliance and side effects.

## Methods

This study was a randomized, double blinded, placebo controlled trial conducted at the Department of Obstetrics and Gynecology, Faculty of Medicine, Chulalongkorn University, Bangkok, Thailand, between May 2019 and May 2020. This study was approved by the Research Ethics Committee of the Faculty of Medicine, Chulalongkorn University. Written informed consent was obtained from all participants. This clinical trial was registered prospectively at ClinicalTrials.gov (Clinical trials registration: NCT 03935152, Date: May 2,2019). All participants gave written informed consent. The methods were performed in accordance with the Declaration of Helsinki. This study adhered to CONSORT guidelines.

Singleton pregnant women aged 18 to 45 years who presented to the labor room with preterm labor with intact membranes between 24 and 34 weeks of gestation were enrolled into the study. Preterm labor was diagnosed as having a regular uterine contraction accompanied by dilatation and/or effacement of the cervix detected by digital examination [4].  The extent of cervical dilatation was ≤ 5 cm and effacement was ≤ 80% in this study. Transvaginal sonography for cervical length and fibronectin were not used in this study. Pregnant woman with fetal or maternal conditions that required immediate delivery (i.e., placenta previa, severe preeclampsia, abruptio placenta, chorioamnionitis, and fetal distress), fetal anomalies, cervical dilatation of 5 centimeters or more, known allergies to progestogen and contraindications to tocolytic drugs were excluded. Women with a history of preterm birth in this study were not using progesterone. 

Uterine contraction and cervical changes were recorded. Baseline investigations such as complete blood count, urinary analysis, cervical swab culture as well as ultrasonography to evaluate the estimated fetal weight were performed. All participants received a total of 4 doses of dexamethasone (6 mg intramuscularly every 12 hours) and 48 hours of tocolytic. Tocolysis was started with oral nifedipine. Ten milligrams of nifedipine was administered every 15 minutes (total three doses) and 10–20 mg every 6 hours thereafter. If the uterine contraction persisted, then the regimen was changed to intravenous terbutaline (5 to 10 mg/min administered intravenously) until the uterine activity ceased, the therapy failed or there were unacceptable maternal effects such as tachycardia. If the participants could not tolerate terbutaline, then indomethacin was used. A loading dose of 50 mg of indomethacin was administered followed by 25 to 50 mg of indomethacine every six hours.

The participants were randomized into the treatment or the placebo group using a block-of-four technique. The computer was used to generate random number. The co-investigator, who had no contact with the participants, generated the allocation sequence prior to the initiation of the study. The nurses enrolled and assigned the participants to their respective groups. Drug and placebo were prepared prior to the initiation of the study by a pharmacist who was not involved in the study. Ten milligrams of dydrogesterone tablet was put into a capsule. No drug was put into the placebo capsule.

Opaque envelopes containing 21–42 capsules of dydrogesterone or placebo (identical in size, shape and color) were sequentially labeled. A 21-capsule and 42-capsule opaque envelopes were used for participant who had 1-week and 2-week follow-up, respectively. To ensure randomization, each envelope was distributed in sequential numerical order. Dydrogesterone (Duphaston® 10 mg per tablet; Abbott, Bangkok, Thailand) was assigned to the treatment group and corresponding placebo to the placebo group. At the start of the study, dydrogesterone or placebo was administered with oral nifedipine. Drug dosage was one capsule every 8 hours. Treatment was continued until delivery or 37 weeks of gestation. Treatment assignment was not revealed until data collection was completed. The protocol for follow-up was the same as the previous study [11]. The participant was admitted into the labor room until the uterine contraction ceased. The participant was followed every 2 weeks, until 36 weeks or more. After that, the participant was followed once per week until delivery. The participant was asked to return the envelope containing the medication for capsule count at every visit. Good compliance was defined as no medication in the envelope.

The primary outcome was to evaluate the latency period (the time from the onset of preterm labor until birth). The secondary outcomes were to evaluate the gestational age at delivery, rates of preterm delivery less than 34 weeks and 37 weeks, time to recurrent uterine contraction, pregnancy outcomes, neonatal outcomes, maternal compliance with medication use, and side effects. The following neonatal outcomes were collected: low birth weight, birth weight, Apgar scores, respiratory distress syndrome (RDS), birth asphyxia, intraventricular hemorrhage (IVH), necrotizing enterocolitis (NEC), neonatal sepsis, neonatal intensive care unit (NICU) admission and neonatal death.

The sample size calculation was based on the latency period of the control group (no progesterone supplementation) from a pilot study. The latency period from the pilot study was 22.5 ± 15.9 days. We expected 14 days increase in latency period when dydrogesterone was used. After adjusting for the withdrawal rate of 10 %, a minimum of 24 women is needed for each group to detect statistical difference (α = 0.05, β = 0.2). Thus, a total of 48 participants were required for this study.

### Statistical analysis

Statistical analysis was performed by SPSS version 22 (IBM Corp., Armonk, NY, USA). Chi-squared test and Fisher exact test were used to compare the categorical data. Student’s t test was used to compare the continuous data. The Mann-Whitney U test was used to compare the nonparametric data. An intent-to-treat (ITT) analysis was used for this trial. Statistically significance was defined as *p* value < 0.05.

## Results

**Fig. 1:**
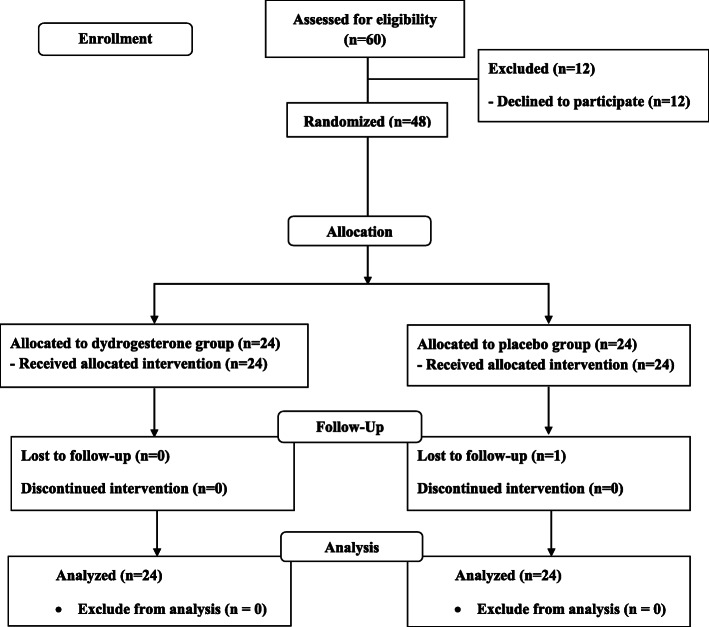
Profile of patient follow-up following randomization to either dydrogesterone or placebo group

Forty-eight women were enrolled into this study. All participants were randomly allocated into the two groups. Twenty four participants received oral dydrogesterone and 24 received placebo. One woman from the placebo group was lost to follow-up (Fig. 1). However, 48 women were included in the ITT analysis. For the demographic characteristics, there were no significant differences between the groups in respect to age, gravidity, parity, gestational age, and pre-pregnancy body mass index (BMI) (Table 1).

**Table 1:** Comparison of selected maternal characteristics of the dydrogesterone group and the placebo group

Characteristic	Dydrogesterone group (*n* = 24)	Placebo group (*n* = 24)	*p* value
Age	29.9 ± 5.7	29.2 ± 7.6	0.703
Gravidity			0.772
Primigravida	13 (54.2 %)	12 (50 %)	
Multigravida	11 (45.8 %)	12 (50 %)	
Parity			0.365
Nulliparous	14 (58.3 %)	17 (70.8 %)	
Multiparous	10 (41.7 %)	7 (29.2 %)	
History of preterm birth	2 (8.3 %)	1 (4.2 %)	1.000
BMI	22.5 ± 4.7	22.2 ± 5.9	0.83
GA at admission (weeks)	31.5 ± 2.1	31.4 ± 2.3	0.804
Median of cervical dilatation (cm)	1 (1-3)	1 (1-3)	0.361

Data presented as mean ± SD, median (range) or n (%). *BMI *Body mass index, *GA *Gestational age

The median latency periods were not significantly different between the dydrogesterone group (27.5 days) and placebo group (34 days, p = 0.45 (Table 2)). There were also no differences in the gestational age at delivery, percentage of preterm delivery before 34 weeks and 37 weeks, and mode of delivery between the two groups. However, the time to the recurrence of contractions among participants who had recurrent preterm labor was significantly longer in the dydrogesterone group than in the placebo group (30.6 ± 12.3 vs. 13.7 ± 5.0, *p* = 0.01). Pregnancy complications were detected in 4 participants: one participant from the placebo group had an endometritis and three participants from the dydrogesterone group had infected wound, postpartum psychosis, and postpartum hemorrhage. When the results for nulliparous group were analyzed, the latency was not different between groups. When the results according to gestational age at admission were analyzed, the latency was still not different between groups.

**Table 2:** Latency periods, gestational age at delivery, mode of delivery, recurrent uterine contraction and pregnancy outcomes

	Dydrogesterone group (*n* = 24)	Placebo group (*n* = 24)	*p* value
Latency period(days)	27.5 (12.5, 37.8)	34 (11, 48)	0.448
GA at delivery	35.4 ± 2.8	36.2 ± 3.0	0.392
GA at delivery < 34 week	7 (29.2 %)	5 (20.8 %)	0.504
GA at delivery < 37 week	16 (66.7 %)	9 (37.5 %)	0.082
Mode of delivery			0.382
Vaginal delivery	15 (62.5 %)	12 (50 %)	
Cesarean delivery	9 (37.5 %)	12 (50 %)	
Time to recurrent uterine contraction (days)	30.6 ± 12.3	13.7 ± 5.0	0.01
Pregnancy complication	3 (12.5 %)	1 (4.2 %)	0.608

Data presented as median (interquartile range), mean ± SD or n (%). *GA *Gestational age

The compliance was good in both groups. There was no difference in the compliance between the two groups (95.8 % vs. 83.3 %, *p* = 0.15 in dydrogesterone group, and placebo group, respectively). Four participants (17.3 %) from the dydrogesterone group had side effects such as tachycardia (*n *= 1), nausea/vomiting (*n* = 1) and hypokalemia (*n* = 2).

Neonatal outcomes are shown in Table 3. Neonatal birth weight, and apgar scores at 1 and 5 minutes did not differ between the two groups. Furthermore, RDS, IVH, NEC, sepsis, apnea of prematurity (AOP), transient tachypnea of newborn (TTNB) and NICU admission were not significantly different between the two groups. There was no neonatal mortality in this study.

**Table 3:** Neonatal outcomes

Characteristic	Dydrogesterone group (*n* = 24)	Placebo group (*n* = 24)	*p *value
Birth weight (grams)	2544.5 ± 734.2	2649.2 ± 674.8	0.614
Apgar score			
At 1 min < 7	2 (8.3 %)	4 (16.7 %)	0.666
At 5 min < 7	0	1 (4.2 %)	1.000
Neonatal complication			
RDS	6 (25 %)	3 (12.5 %)	0.461
IVH	0	0	NA
NEC	0	0	NA
Sepsis	3 (12.5 %)	6 (25 %)	0.461
AOP	1 (4.2 %)	1 (4.2 %)	1.000
TTNB	1 (4.2 %)	1 (4.2 %)	1.000
NICU Admission	7 (25 %)	5 (16.7 %)	0.504

Data presented as mean ± SD or n (%)

*RDS *Respiratory distress syndrome, *IVH *Intraventricular hemorrhage, *NEC *Necrotizing enterocolitis, *AOP *Apnea of prematurity, *TTNB *Transient tachypnea of new born, *NICU *Neonatal intensive care unit

## Discussion

This randomized, double blinded, placebo controlled trial assessed the efficacy of oral dydrogesterone as an adjunctive therapy for maintenance treatment in preterm labor treated with tocolysis and corticosteroids. Maintenance treatment aims to prolong the latency periods until delivery, therefore it will reduce the rate of preterm birth. Latency period in this study was not different between the dydrogesterone group and the placebo group. Additionally, gestational age at delivery, preterm delivery before 34 and 37 weeks, pregnancy outcomes and neonatal outcomes were also not different in both groups. However, the time to the recurrence of uterine contractions in participants with recurrent preterm labor was longer in the dydrogesterone group.

In this study, the latency periods were not different between the dydrogesterone group and the placebo group. This result was consistent with a prior study that assessed the effect of 20 mg of oral dydrogesterone on latency periods [11]. In a previous meta-analysis by Eke et al. [15], randomized controlled trials that used oral progesterone,vaginal progesterone, or 17-hydroxyprogesterone caproate found no significant differences in latency periods and no significant reduction in the rate of preterm birth. Comparable results were found in the study by Wood et al. [16] that found vaginal progesterone treatment did not increase the mean latency to delivery. Previous studies reported that progestogen use could prolong latency periods [7–9]. These studies used vaginal progesterone [7, 8], while another study used oral micronized progesterone [9]. In contrast, our study did not show that dydrogesterone could prolong latency periods. This difference may be due to the difference in the type of progesterone used and the route of drug administration used.

Progesterone is a key factor in maintaining the quiescence phase during pregnancy and its withdrawal resulting in parturition [3], however the mechanism is not well understood. Progesterone inhibits myometrial contraction through various mechanisms; it can regulate the expression of progesterone receptors, interfere with the regulation of corticotrophin-releasing hormone, and block proinflammatory cytokine [5]. In vitro studies [13, 17] found that progestogens can inhibit spontaneous myometrial contractility but this was dependent on the dose used. In another study, only dydrogesterone could inhibit spontaneous myometrial contractility compared to other progestogens [13]. As for our study, the duration until recurrent contraction in participants that had recurrent preterm labor was significantly longer in the dydrogesterone group. Despite that, gestational age at delivery, pregnancy outcomes, and neonatal outcomes were not different between the two groups.

The percentage of preterm delivery before 34 and 37 weeks in this study were not different between the two groups. This finding was similar to previous studies [12, 18] that showed the rates of preterm delivery less than 34 and 37 weeks were not significantly reduced when vaginal progesterone was used compared to the placebo group. On the other hand, another study [19] found that vaginal progesterone after tocolysis in threatened preterm labor could reduce preterm delivery before 34 weeks. This discrepancy may be due to the difference of the study population and the route of progesterone administration. For our study, we used oral dydrogesterone in preterm labor whereas Bomba-Open, et al. used vaginal progesterone in threatened preterm labor.

The strength of this study was its design. This was a randomized, double blinded, placebo-controlled trial. Oral dydrogesterone was selected for this study because of its good oral bioavailability and oral administration. The dosage of 30 mg per day (10 mg t.i.d. po pc) was chosen because it covered its half life. The limitation of this study was that various tocolytic drugs were used. This may be used in clinical practice. Tocolysis is tailor-made for each pregnant woman. Additional study is needed to assess the benefits of other progestogen as an adjunctive treatment in preterm labor. Another limitation was that the power calculation used quite substantial and generous assumption, not close to real-life, and was underpowered. 

## Conclusions

Adjunctive treatment with 30 mg of oral dydrogesterone per day could not prolong the latency periods in preterm labor when compared to the placebo. Oral dydrogesterone could prolong the time to the recurrence of uterine contractions in participants who had recurrent preterm labor. There were no differences in gestational age at delivery, percentage of preterm delivery before 34 and 37 weeks, pregnancy outcomes, neonatal outcomes, compliance and side effects between the two groups.

## Data Availability

The ethical approval did not include permission to disclose the data publicly. It can be made available by the authors upon reasonable request.

## Abbreviations

RDS: Respiratory distress syndrome
IVH: Intraventricular hemorrhage
NEC: Necrotizing enterocolitis
ACOG: American College of Obstetricians and Gynecologists
ITT: Intent-to-treat
BMI: Body mass index
NICU: Neonatal intensive care unit
AOP: Apnea of prematurity
TTNB: Transient tachypnea of new born
